# Platelet-rich plasma, a biomaterial, for the treatment of anterior talofibular ligament in lateral ankle sprain

**DOI:** 10.3389/fbioe.2022.1073063

**Published:** 2022-12-22

**Authors:** Jieyuan Zhang, Cheng Wang, Xueqian Li, Shaoling Fu, Wenqi Gu, Zhongmin Shi

**Affiliations:** ^1^ Department of Orthopedic Surgery, Shanghai Sixth People’s Hospital, Shanghai, China; ^2^ Department of Orthopedic Surgery, Shanghai Sixth People’s Hospital East Campus, Shanghai, China

**Keywords:** anterior talofibular ligament, platelet-rich plasma, lateral ankle sprain, signal/noise ratio, magnetic resonance imaging, biomaterials, locomotor system

## Abstract

**Background:** Because of the rising prevalence of anterior talofibular ligament (ATFL) damage, there is a considerable interest in developing innovative techniques to improve the biological healing response of ATFL. Platelet-rich plasma (PRP) includes several growth factors linked to a favorable healing response, however none of the studies involved both quality evaluation and clinical results to evaluate this treatment.

**Purpose:** To determine the clinical results of PRP injections into the ATFL in lateral ankle sprain (LAS) patients, as well as the quality of the ATFL based on radiographic outcomes.

**Methods:** Patients with clinically confirmed grade II LAS for the first time (*n* = 83) were examined. The clinical outcomes of three types of injection methods were evaluated: none, once within 48 h after the sprain, and once more 4 weeks later after first injection. PRP was injected into the tear site of the ATFL using ultrasound guidance, and all ankles were immobilized for 2 weeks. The American Orthopedic Foot and Ankle Score (AOFAS) ankle-hindfoot scale and the Visual Analogue Scale (VAS) were used to assess the results at 2, 6, 8, 24, and 48 weeks of follow-up. The signal/noise ratio (SNR) value of Magnetic Resonance Imaging (MRI)-based ATFL signal intensity can disclose ATFL quality. The ATFL SNR results were then assessed 8, 24 and 48 weeks following the first injection to compare ATFL quality.

**Results:** The PRP injection groups outperformed the control group in terms of clinical outcomes, and the two injections group outperformed other groups in terms of pain reduction and functional outcome at 8 weeks. The clinical results of all groups were comparable at 6 and 12 months follow-up. ATFL SNR findings improved significantly across all groups over time. At the same follow-up time, the PRP injection groups outperformed the control group, and the best SNR result showed in the two injections group at the final follow-up.

**Conclusion:** PRP injection helped relieve early symptoms of LAS, although all patients saw a similar development after 6 months. The two PRP injections group produced considerably better clinical results and quality of the ATFL in short-term follow-up.

## Introduction

The most frequent musculoskeletal injury in the physically active group is lateral ankle sprain (LAS) (Colville et al., 1992), it is also a prevalent problem in the general population (Doherty et al., 2014). It is worth mentioning that the anterior talofibular ligament (ATFL) is the most commonly injured ligament structure and, in some circumstances, the only ligament structure that is damaged in LAS (Malliaropoulos et al., 2006; Fong et al., 2009). Recent studies have also revealed that the ATFL is the primary anatomical component responsible for lateral ankle stability, and that ATFL repair/reconstruction alone is adequate to restore lateral ankle stability (Takao et al., 2016; Ko et al., 2020).

Clinically, ankle sprains are classified into three grades based on the severity of the injury: grade I, in which there are no joint instability or macroscopic ruptures yet there is a minor stretching of the ligaments; grade II (moderate), in which the ligaments are partially torn, there is significant discomfort and inflammation, and the patients have difficulties supporting themselves; and grade III, in which the ligaments have fully burst, resulting in extreme pain, edema, and hemorrhage, as well as incapacity to function due to instability (Wolfe et al., 2001; Petersen et al., 2013). For all grades of LAS, conservative management and early functional rehabilitation remain the mainstay of therapy. Platelet-rich plasma (PRP) is an autologous derivative of whole blood that causes a supra-physiological release of growth factors in chronic injuries to accelerate recovery. Platelet alpha granules produce growth factors, which cause chemotaxis, cell migration, angiogenesis, proliferation, differentiation, and matrix synthesis. PRP is gradually making its way to the forefront of sports and rehabilitation medicine, and its involvement in many soft injuries has recently become the focus of study (Huang et al., 2020; Andrade et al., 2021; Hohmann et al., 2021).

Despite its popularity, little was understood about PRP research’s significance in LAS treatment. Lai et al. described a case of LAS with a complete rupture of the ATFL that healed completely and resulted in early ankle stability following PRP (Lai and Sit, 2018). A similar study demonstrated the efficacy of PRP for the treatment of moderate ankle sprains, with the PRP injection group showing the greatest reduction in pain and better functional scores than the control group at 8 weeks, though a similar evolution was seen in patients treated with or without PRP after 24 weeks (Rivera et al., 2020). However, these studies only looked at the clinical results of PRP on ankle sprains, not the impact of PRP on ATFL quality. A recent consensus paper stated that surgeons should consider ligament quality while deciding surgical technique (Michels et al., 2018). As a result, the optimal therapy should not only have a positive therapeutic impact but also restore the quality of the ligament. Several studies have found a link between the signal to noise ratio (SNR), which indicates the signal strength of the ATFL on Magnetic Resonance Imaging (MRI), and the condition of the ATFL (Liu et al., 2017). They discovered that ankles with chronic instability had a higher ATFL SNR than healthy stable ankles, and that the mean ATFL SNR value decreased after repair surgery, implying that the ATFL SNR could be used to assess ligament tissue quality (Li et al., 2017; Li et al., 2019). Ahn et al. also demonstrated that the SNR was more closely connected to ATFL quality than the presence of a tear or the degree of strain (Ahn et al., 2021).

The goal of this study was to evaluate the clinical effect of PRP in patients with acute LAS using a foot and ankle function and pain scale, as well as to investigate ATFL quality with SNR value in a short-term follow-up.

## Methods

### Patients and experiment design

From August 2019 to October 2020, patients diagnosed with acute LAS according to the standards of the American College of Foot and Ankle Surgeons were included in this prospective research. Patients were eligible if they were 18–65 years old, with first-time LAS lasting no more than 48 h, grade II, and mild to moderate instability. Patients who were pregnant, had previous ankle procedures or therapies, or had other ankle-related disorders were excluded. All participants provided written informed consent, and the study was approved by the Ethics Committee of Shanghai Sixth People’s Hospital.

Patients were divided into three groups according to three types of PRP injection method, none (control), once within 48 h after the sprain (one injection), and once more 4 weeks later after first injection (two injections). The foot of control group was put in a short plaster cast in a neutral position. Patients were instructed to bear weight as soon as the discomfort permitted. In the PRP injection groups, PRP was previously provided to the ATFL, and then a short plaster cast with the foot in neutral position was employed in the same way. The plaster cast was removed from all groups of patients after 2 weeks of therapy, and patients got the identical rehabilitation routine after the cast was removed.

All patients were examined to identify their degree of pain using the Visual Analogue Scale (VAS). The American Orthopedic Foot and Ankle Score (AOFAS) score was used to evaluate all of the patients at 2, 6, 8, 24 and 48 weeks. The MRI-based ATFL SNR value was used to quantitatively compare the ATFL quality. All radiographic parameters and clinical outcomes were measured by two orthopaedic surgeons.

### MRI assessment

In a neutral stance, a 3.0-T MRI scanner (MAGNETOM Verio, A Tim System; Siemens) was used to scan the ankles of the patients. All patients had the same scan sequence and MRI settings. The MRI acquisition protocol included fat-suppressed proton density-weighted (FS PDW) turbo spin-echo (TSE) Dixon sequences (slice thickness = 2.5 mm, TR/TE = 4,960/38 ms) in the coronal planes, FS PDW TSE Dixon sequences (slice thickness = 2.5 mm, TR/TE = 3,500/35 ms) in the sagittal planes, and T2-weighted TSE sequence For all images, the field of view was 150 mm × 150 mm and the matrix was 300 × 300. Using axial T2-weighted MRI sequences, the ATFL was examined. On T2-weighted images, it often appears as a linear structure with low signal intensity. The signal intensity was calculated at both the ATFL site and a background site (about 2 cm away from the ATFL) with a region of interest. The SNR was calculated using the following equation to quantify the normalized signal intensity of the ATFL: SNR = ATFL signal/background signal. For each case, two slices were typically chosen for SNR measurements, which were then averaged to determine the final SNR value. The diagnostic SNR cutoff point for distinguishing between normal and abnormal but reparable ATFL was 11.2, and it took 32.3 to distinguish between an irreparable and reparable ATFL (Ahn et al., 2021).

### PRP preparation and application

PRP was made utilizing a commercially available product (WEGO Platelet-Rich Plasma Preparation Kits, WEGO Ltd. Shandong, China), which generated a platelet concentration factor of more than six times that of whole blood and an estimated platelet recovery rate of 80 percent. The concentration of leukocytes in this PRP is no more than four times that of whole blood. Each patient had 40 ml of whole blood collected into anti-coagulant-treated blood collection tubes. A nurse utilized the WEGO PRP Kits to collect 3-4 ml PRP (first time: 800 g 10 min, second time: 1100 g 10 min) at room temperature. Prior to the PRP injection, 0.5 ml of 10% calcium chloride solution was administered to activate the PRP. The PRP was then injected into the tear site of the ATFL using ultrasound guidance.

### Statistical analysis

All statistical analysis was conducted using SPSS 24.0 in this study, and all data were tested for normality. Normally distributed continuous variables are shown as the mean ± SD, otherwise data were expressed as median and interquartile range. ANOVA and t-test were performed for normally distributed continuous variables. And non-normally distributed continuous variables were compared using the Wilcoxon test. The χ2 test was used to evaluate differences in categorical data. *p* < 0.05 was considered statistically significant.

## Results

23 patients received none PRP injection, 27 patients received one PRP injection, and 22 patients received two PRP injections. Demographic information of three groups is summarized in Table 1. The baseline characteristics of the three groups in terms of age, gender, BMI, and sprain side were examined.

**TABLE 1:** Patient demographics characteristics^a^.

Characteristic	Control	One	Two	*p*-Value
Number of patients	23	27	22	—
Age (years)	31.2 ± 7.9	31.9 ± 8.2	31.5 ± 8.1	0.9523
Gender (Male/Female	12/11	13/14	13/9	0.745
BMI (Kg/m^2^)	24.7 ± 3.0	26.0 ± 2.9	25.2 ± 3.3	0.3438
Side of sprain (Right/Left)	9/14	11/16	12/10	0.516

^a^ Data are presented as mean ± standard deviation (SD). BMI, body mass index.

The comparison of the baseline characteristics of the three groups was statistically insignificant.

In VAS score, during the initial patient evaluation, all groups experienced significant pain, with no differences among groups. Patients who had PRP injections, however, had considerably larger pain reduction since their initial evaluation and throughout the trial period compared to the control group, despite the fact that all groups had comparable outcomes in the last two follow-ups. Notably, the pain reduction after the second injection was greater in the two injections group than in the one injection group. At 8 weeks, the two injections group had the greatest reduction in pain compared to other groups (Table 2).

**TABLE 2:** Clinical results of the visual analogue scale (VAS) score^a^.

Follow-up (weeks)	Control	One	Two	*p*-Value
Pre-treatment	7.4 ± 1.1	7.4 ± 1.0	7.5 ± 1.0	0.9774
Two	6.3 ± 0.7	5.0 ± 0.9	5.0 ± 1.1	<0.0001
Six	4.1 ± 0.8	2.6 ± 0.6	2.0 ± 0.7	<0.0001
Eight	2.0 ± 0.6	0.9 ± 0.7	0.4 ± 0.5	<0.0001
Twenty-Four	0.3 ± 0.4	0.1 ± 0.3	0.1 ± 0.3	0.10
Forty-Eight	0.1 ± 0.3	0.1 ± 0.3	0.1 ± 0.2	0.86

^a^ Data are presented as mean ± standard deviation (SD).

According to the AOFAS scores, all patient groups improved during the trial. During the follow-up period, the improvement in AOFAS scores was sustained. When we compared the three groups, we discovered that the improvement in patients treated with PRP was much greater than in individuals who did not receive PRP injection. After the second PRP injection, AOFAS values improved significantly and showed no significant difference between 8 weeks and the last follow-up. Furthermore, in the last two follow-ups, the AOFAS values were comparable in all groups (Table 3).

**TABLE 3:** Clinical results of the American orthopedic foot and ankle score (AOFAS) ankle-hindfoot scale^a^.

Follow-up (weeks)	Control	One	Two	*p*-Value
Two	78.2 ± 4.2	84.2 ± 2.5	84.5 ± 2.5	<0.0001
Six	84.0 ± 2.5	87.7 ± 2.2	91.7 ± 2.2	<0.0001
Eight	87.3 ± 2.0	92.1 ± 2.6	96.4 ± 2.1	<0.0001
Twenty-Four	98.1 ± 1.2	98.7 ± 0.9	98.8 ± 1.0	0.0866
Forty-Eight	99.1 ± 1.1	98.4 ± 0.9	99.5 ± 0.7	0.2965

^a^ Data are presented as mean ± standard deviation (SD).

All patient groups experienced a progressive decline in ATFL SNR values. The SNR values in the PRP injection groups considerably dropped over the course of the follow-up. SNR values significantly dropped after the second PRP injection. The SNR values of each group at the last follow-up were still significantly different, in contrast to clinical outcomes like VAS and AOFAS. The group receiving twice PRP injections had the lowest SNR score (Table 4).

**TABLE 4:** MRI-based ATFL SNR value^a^.

Follow-up (weeks)	Control	One	Two	*p*-Value
Eight	34.9 ± 5.5	28.4 ± 4.5	21.9 ± 5.1	<0.0001
Twenty-Four	21.9 ± 3.5	15.8 ± 2.4	13.0 ± 1.9	<0.0001
Forty-Eight	17.5 ± 2.9	11.9 ± 1.7	9.2 ± 1.4	<0.0001

^a^Data are presented as mean ± standard deviation (SD).

## Discussion

Lateral ligament insufficiency secondary to LAS is one of the most frequently encountered musculoskeletal injuries, and −73% are related to the ATFL. The ATFL injury increases the likelihood of repeated sprains and anxiety during inversion stress. For all grades of LAS, conservative management and early functional rehabilitation remain the mainstay of therapy (Doherty et al., 2017). PRP has been described for the treatment of ankle sprains in high-performance athletes as well as the general population. In the study by Juancarlos et al., the use of PRP therapy as an adjuvant for the treatment of lateral ankle sprains results in less discomfort and a better functional outcome when compared to immobilization alone (Rivera et al., 2020). However, the functional scales assessed are based only on the perspective of the patient and might provide subjective findings; also, no radiographic evaluation (MRI or ultrasound) of the influence of PRP on ATFL quality was done. Another study described a case of LAS with full ATFL rupture that exhibited complete ligament repair and early ankle stability following PRP. The healing is supported by dynamic ultrasound images and MRI. They indicated that in the future, PRP might be used as an alternative non-surgical treatment option in LAS, with the potential to avoid the development of CAI and post-traumatic ankle osteoarthritis (PTOA) (Lai and Sit, 2018). However, the evidence in this study was limited in quantity, there were no placebo groups, and only radiographically proven ligament restoration was available, with no functional scores. The goal of this study was to assess the impact of PRP in acute sprain, both functionally and radiographically, and to see if ATFL quality correlated with functional scores.

MRI is a great non-invasive method for measuring and evaluating ATFL dimensions or signal intensity when compared to other technologies (Dimmick et al., 2008; Katier et al., 2015). In general, undamaged ATFLs provide low signal intensity on MRI, whereas injured ATFLs produce high signal intensity. Liu et al. previously used MRI to assess the signal/noise ratio (SNR) of the repaired ATFL (Liu et al., 2017). They discovered that ankles with chronic instability had a greater ATFL SNR than healthy stable ankles, and that the mean SNR value of the ATFL dropped following repair surgery, implying that the ATFL SNR might be used to assess ligament tissue quality. Li et al. performed arthroscopic examinations on 60 CAI patients and matched their findings to MRI scans to confirm ATFL rips (Li et al., 2019). Cases with thick ATFL remnants and excellent tension were classified as having good ATFL, whereas those with thin ATFL remnants and significant laxity were classified as having poor ATFL. According to their findings, poor ATFL conditions were indicated by an SNR value of 10.4. Based on these findings, data from a cohort of 70 patients who underwent the modified Broström procedure were examined for clinical outcomes at an average follow-up of 46 months. The results revealed that the good ATFL condition group had a higher rate of return to sports than the poor ATFL condition group did, who had higher SNR values. According to ATFL tension and quality, Ahn et al. also demonstrated that a low SNR evaluated by preoperative MRI predicted better ATFL reparability and that the SNR had predictive value to assess the ATFL reparability utilizing the arthroscopic all-inside ATFL repair procedure (Ahn et al., 2021). They confirmed that the diagnostic SNR cutoff point for differentiating between an aberrant but repairable ATFL and a normal ATFL was 11.2, and that it was 32.3 for differentiating between an irreparable ATFL and a repairable ATFL. But there have been no reports of quality evaluations following PRP therapy for ATFL in LAS. The AOFAS and VAS scores of the PRP injection groups improved significantly more than those of the control group. Additionally, there was no difference in the two scores at the 24- and 48 weeks follow-ups. The SNR values of the three groups all continued to decline, with the decline of the PRP injection groups being more pronounced.

Repeated PRP injections have been shown in several trials to increase the efficacy and lengthen the therapeutic impact in specific conditions (Huang et al., 2017; Chouhan et al., 2019; Yasui et al., 2021). There is not yet a publication in PRP comparing the various injection protocols for treating ATFL in LAS. This is the first clinical research to examine the various PRP injection procedures used to treat ATFL, and MRI was used to analyze the quality of the ATFL. Our study also demonstrated that two PRP injections group had the greatest reduction in pain and best AOFAS score at 8 weeks follow-up, and the SNR value had dropped to normal cutoff point at 48 weeks follow-up. We take out the other PRP therapy factors in order to critically examine the impact of various PRP injections. The identical PRP Kit, preparation technique, concentration, and pre-activation were administered to all patients. All patients received the same injection technique, volume and injection protocols. This research shows the longest follow-up in patients with lateral ankle sprain treated with PRP that we are aware of.

There are limitations of this study. First, a clinical classification—which varies depending on the observer—was used to make the diagnosis of LAS. Second, the ATFL has not been seen morphologically to support the effects of PRP injection. Third, the amount of time available for evaluating ligament quality was limited. Finally, the MRI SNR was not performed when the patient was first evaluated. The time and number of PRP injections required to return the ATFL to its ideal state have still to be determined.

## Conclusion

In comparison to immobilization alone, PRP injection appears to be more successful in LAS in short-term follow-up and enables the patient to report less pain during his recovery period and a better functioning outcome. Additionally, the group receiving two PRP injections seems to have improved clinical results and ATFL quality recovery.

## Data Availability

The original contributions presented in the study are included in the article/Supplementary Material, further inquiries can be directed to the corresponding authors.
